# Materia Medica and Therapeutics

**Published:** 1896-02

**Authors:** J. S. Cassidy

**Affiliations:** Covington, Ky.


### Materia Medica and Therapeutics.

BY J. S. CASSIDY, M.D., D.D.S., COVINGTON, KY.

Abstract of paper read at American Dental Association, August, 1895.

One of the queer things appearing with the demand for iodoform substitutes is that they must all be more or less rich in iodin. Aristol, for instance, is thymol iodid, iodoform being menthenyl iodid. It does not follow, however, that these must give up their iodin in order to be therapeutically active. Indeed, comparatively few chemical compounds are dependent on decomposition for their medicinal activity, among such being the peroxides of hydrogen and of sodium, the hypochlorites, etc.

The disinfectants, as a rule, either give to or take from the infectious matter their individual equivalent radicals, negative or positive, as the case may be. Antiseptics, on the contrary, do not, as a class, lose their chemical identity by contact with organized material; of such are iodoform, common salt, carbolic acid, etc. They act as antiseptics in their capacity of compounds, as is easily proved by sufficient experiments, notwithstanding some authorities claim that iodoform, at least, is effective only by setting its iodin free.

Many of the most dangerously active bodies in their influence on animal life retain their compound nature when in action, of which prussic acid and arsenious oxide will serve as examples. We need, therefore, not necessarily pay attention to the relative proportion of any element in a compound so far as the medicinal effects are concerned, unless the mode of action of the compound involved is by co-incident decomposition in the presence of organized structure, and of such iodoform and its cogeners are not good examples.

One of the newest drugs belonging to this category is called di-iodoform. When presented in powder to freshly-cut or abraided surfaces, or in chloroform solution into root-canals, it fulfills the duties of a very good antiseptic, and when either in crystal or powder it is inserted into root-canals and fused by a hot broach. It resolves, on cooling, into an insoluble, black, concreted mass of permanent character. * *

Electrozone has proven to be an acceptable, inexpensive and effective germicide and disinfectant. * * The 25 percent dilution of electrozone does good service as an injection into the antrum immediately after the withdrawal of pus. I think it is preferable for this purpose to peroxide of hydrogen, inasmuch as it is not accompanied by a too rapid evolution of gas and consequent pressure in the cavity.

Many experiments made during the past year in the laboratory and on the living subject as well, with aqueous solutions of the gas formaldehyd, have convinced the writer that as an embalmer of pulp debris, it is superior to all other agents thus far employed exclusively for such purposes. The aldehyd condenses into paraldehyd in the meshes of the devitalized tissue, and at the same time absorbs the organic laden moisture present, changing the debris into a hard, coherent, non-infectious residue.

In closing this report I wish to thus publicly thank Dr. Harlan for having called our attention to the virtues of trichloracetic acid. When properly diluted and applied to the inflamed parts, including and surrounding the peridental membrane, due to whatever cause, its magical influence, owing to its solvent, caustic, stimulant and astringent action combined, in lessening, if not curing the disease, is so evident, that a specific position has been justly awarded it by those who have given it a fair and impartial trial.

